# Bacteriophage therapy for multidrug-resistant infections: current technologies and therapeutic approaches

**DOI:** 10.1172/JCI187996

**Published:** 2025-03-03

**Authors:** Minyoung Kevin Kim, Gina A. Suh, Grace D. Cullen, Saumel Perez Rodriguez, Tejas Dharmaraj, Tony Hong Wei Chang, Zhiwei Li, Qingquan Chen, Sabrina I. Green, Rob Lavigne, Jean-Paul Pirnay, Paul L. Bollyky, Jessica C. Sacher

**Affiliations:** 1 Division of Infectious Diseases and Geographic Medicine, Department of Medicine, Stanford University, Stanford, California, USA.; 2 Department of Medicine, Yale University, New Haven, Connecticut, USA.; 3 Division of Public Health, Infectious Diseases and Occupational Health, Mayo Clinic College of Medicine, Rochester, Minnesota, USA.; 4 Laboratory of Gene Technology, Department of Biosystems, KU Leuven, Leuven, Belgium.; 5 Laboratory for Molecular and Cellular Technology, Queen Astrid Military Hospital, Brussels, Belgium.; 6 Phage Directory, Atlanta, Georgia, USA.

## Abstract

Bacteriophage (phage) therapy has emerged as a promising solution to combat the growing crisis of multidrug-resistant (MDR) infections. There are several international centers actively engaged in implementation of phage therapy, and recent case series have reported encouraging success rates in patients receiving personalized, compassionate phage therapy for difficult-to-treat infections. Nonetheless, substantial hurdles remain in the way of more widespread adoption and more consistent success. This Review offers a comprehensive overview of current phage therapy technologies and therapeutic approaches. We first delineate the common steps in phage therapy development, from phage bank establishment to clinical administration, and examine the spectrum of therapeutic approaches, from personalized to fixed phage cocktails. Using the framework of a conventional drug development pipeline, we then identify critical knowledge gaps in areas such as cocktail design, formulation, pharmacology, and clinical trial design. We conclude that, while phage therapy holds promise, a structured drug development pipeline and sustained government support are crucial for widespread adoption of phage therapy for MDR infections.

## Introduction

Antimicrobial resistance (AMR) poses a critical global health threat that necessitates innovative therapeutic approaches (1, 2). Bacteriophages (phages), viruses that infect and destroy bacteria, have emerged as a promising therapeutic solution to combat multidrug-resistant (MDR) infections (3, 4).

Phage therapy, a concept that originated in the early 20th century (5), was largely abandoned in Western Europe and North America following the introduction of antibiotics in the 1940s, although its use continued in Eastern Europe (6). However, the growing AMR crisis has rekindled widespread interest in this therapeutic modality, with numerous successful cases reported worldwide (7). Personalized phage therapy, which involves selecting and optimizing phages for individual cases, is now being refined at several centers across Europe, the United States, and Australia.

Recent studies have demonstrated the efficacy of phage therapy in treating MDR infections. A recent systematic review of 59 phage therapy studies published between 2000 and 2020 found that 78.8% of 1,904 patients who received compassionate phage therapy experienced clinical improvement, and pathogen eradication was achieved in 86.7% of cases (8). Similarly, a retrospective case series of 100 consecutive phage therapy cases reported clinical improvement in 77.2% of cases and pathogen eradication in 61.3% (9). These findings, along with those of several in-depth, recent review articles, highlight the potential and limitations of phage therapy in the ongoing battle against MDR infections (3, 10–15).

This Review seeks to focus on the technical aspects of current phage therapy practices, with a particular emphasis on technology development and clinical applications. It also examines the development of phage therapy products and protocols from the perspective of the conventional drug development pipeline, providing a road map for future research and clinical translation efforts.

## Phage preparation and administration

The implementation of phage therapy involves multiple steps, from phage sourcing and characterization through manufacturing, quality control (QC), therapeutic administration, and clinical monitoring. While not all steps are universally applied in every phage therapy, this section outlines the key stages in preparing and delivering phage therapy.

## Phage identification and selection

### Phage sourcing, storage, and characterization.

**Figure 1:**
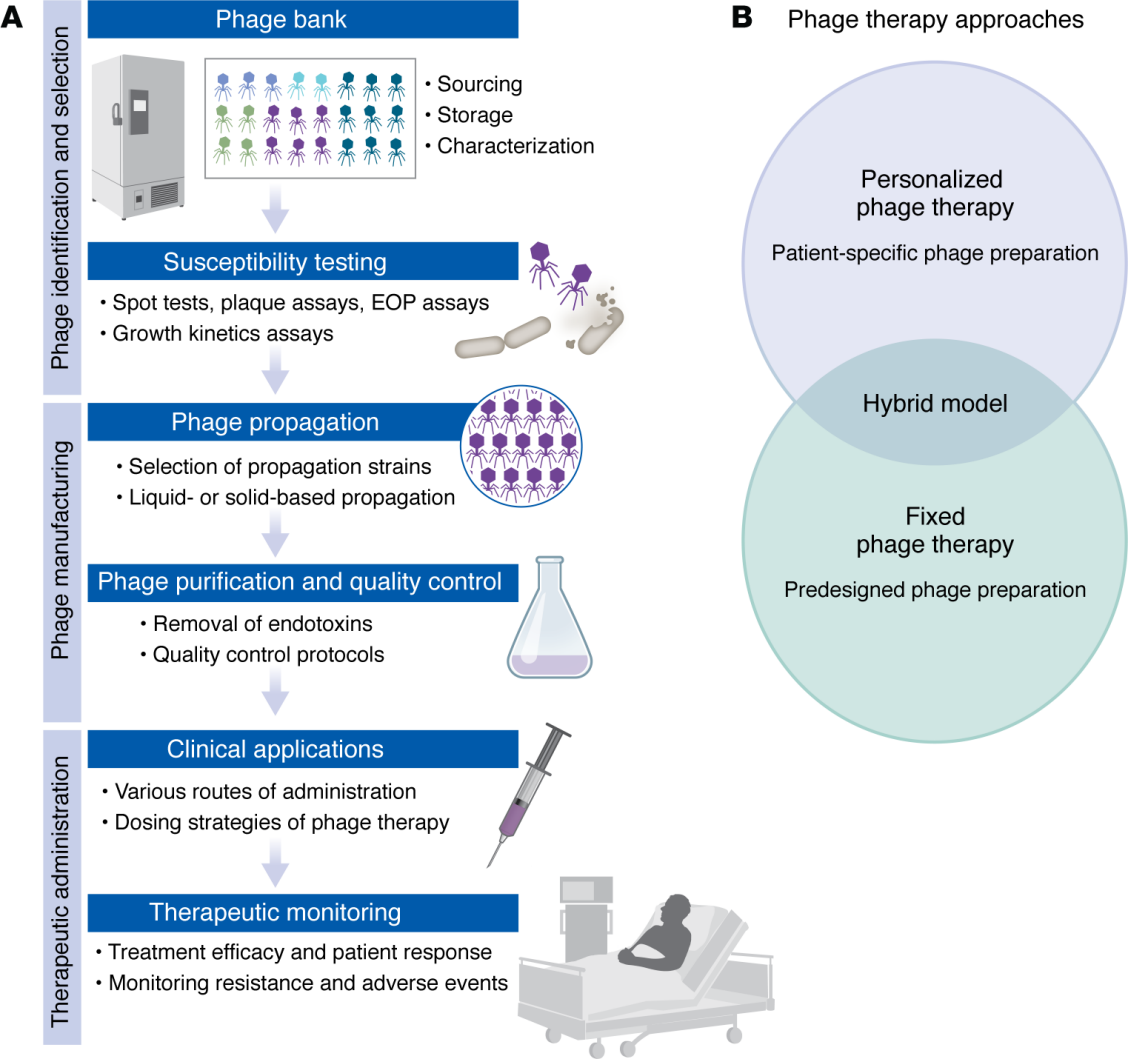
Development and implementation of phage therapy. (**A**) A summary of the key steps in phage therapy development and clinical implementation. The process typically begins with phage identification and selection, including phage bank establishment (sourcing, storage, and characterization of phages), followed by susceptibility testing (using spot tests, plaque assays, efficiency of plating [EOP] assays, and growth kinetics studies). The manufacturing phase involves phage propagation (using selected bacterial strains in liquid- or solid-based systems) and rigorous purification with quality control measures (including endotoxin removal and standardized quality protocols). The therapeutic administration phase encompasses clinical applications (considering various administration routes and dosing strategies) and therapeutic monitoring (tracking treatment efficacy, patient response, and monitoring for potential resistance development and adverse events). Note that these steps are not universally applied in all phage therapies. (**B**) Phage therapy approaches can be personalized to individual patients (patient-specific phage preparation), fixed (preformulated), or administered as a hybrid of the two approaches. The hybrid model represents an intermediate approach combining elements of both personalized and fixed phage therapy strategies.

Phage banks serve as essential repositories of diverse phages for therapeutic and research purposes, ensuring long-term viability and swift access when needed (Figure 1A) (16, 17). These banks, such as the Eliava Institute, the Israeli Phage Bank, the Félix d’Hérelle Reference Center, the Leibniz Institute (DSMZ), and the Phage Australia Biobank, employ various storage methods (18, 19). Common techniques include storage in buffer or growth media at 4°C, cryopreservation in glycerol at –80°C or liquid nitrogen (either with or without host cells), and lyophilization for room temperature or cold storage (19, 20). The most accessible and cost-effective method is 4°C storage, typically using standard phage preservation media such as SM buffer (100 mM NaCl, 8 mM MgSO_4_, 50 mM Tris-HCl, pH 7.5) or the original sterile-filtered growth media. Lyophilization, while potentially causing initial titer loss, offers advantages for long-term storage and transport by freeze-drying in vacuum-sealed vials, often with stabilizing additives like sucrose or polymers (21–26). To further minimize titer loss for long-term storage, some facilities also preserve phages within bacterial cells by freezing down cells shortly after phage infection but before lysis occurs (27, 28). Storage stability varies among phages with phage morphology potentially playing a crucial role. The tailed phages, particularly myoviruses, generally demonstrated superior stability (29). Depending on storage conditions and phage type, viability can range from months to over 32 years (27, 30).

Characterization of banked phages typically includes morphological examination through transmission electron microscopy or cryogenic electron microscopy, receptor identification via mutant libraries and surface-molecule competition assays, and host range determination using plaque assays (31–33). Additional analyses include whole-genome sequencing using next-generation platforms, biofilm inhibition assessment, and regular monitoring of storage stability through titer measurements over time under different conditions.

Effective management of phage banks requires multiple storage sites, robust backup systems, access controls, and efficient inventory tracking to ensure the reliability and accessibility of phage stocks for therapeutic applications (34, 35).

### Phage susceptibility testing.

Phage susceptibility testing is a crucial step in selecting phages with activity against target bacteria (Figure 1A). It identifies phages for clinical use and guides on dosing and administration strategies (36). Phage susceptibility is determined by complex molecular interactions between the phage and host throughout the infection cycle, including phage receptor-binding proteins, host surface receptors, intracellular defense mechanisms, and phage lifestyle (i.e., either lytic or lysogenic) (37–40). Most current therapies use strictly lytic Caudovirales, particularly myoviruses and siphoviruses, owing to their broader host ranges and enhanced stability (13). While podoviruses are less commonly employed, select members of this family have demonstrated therapeutic efficacy (13).

Bacterial cultures from a patient are tested against phages using various in vitro culture-based techniques (41, 42). “Spot tests” apply phage droplets to bacterial lawns to observe zones of inhibition after overnight incubation. “Plaque assays” use serially diluted phage samples to observe countable individual plaques. Plaque assays are essential for confirming productive infection, as they distinguish true virulent activity from nonproductive lysis phenomena such as “lysis from without” (36, 43, 44). “Efficiency of plating (EOP) assays” provide quantitative measurements of phage lytic activity by comparing its performance on test strains relative to a reference host (43, 45). Higher EOP values may suggest potential new propagation hosts, though adoption requires careful consideration of growth characteristics, safety profiles, yield consistency, and purification efficiency, especially for therapeutic applications. “Growth kinetics assays” complement these methods by monitoring bacterial growth inhibition in real-time through optical density measurements. When results differ between plaque formation and growth kinetics, each assay provides complementary information: plaque assays confirm productive infection cycles, while growth kinetics reveal killing rates and resistance development patterns (36). These methods are also employed to evaluate phage-antibiotic and phage-phage interactions during cocktail design, as discussed in detail below.

Recent technological advances include automated optical density measurement systems (46–48), hydrogel-embedded “ready-to-screen” plates (49), tablet-embedded ATP release assays (50), and automated phage plaque image analysis software (51). However, the field continues to lack universally accepted and rapid susceptibility tests (36, 43, 52, 53). This limitation stems from fundamental challenges, including the potential disconnect between in vitro assay results and in vivo conditions (particularly regarding bacterial biofilms within the host) and the absence of standardized criteria for categorizing bacterial isolates as “susceptible,” “intermediate,” or “resistant.” (54). These factors can substantially impact the assessment and prediction of phage therapy efficacy.

Efforts to establish phage susceptibility testing standards are ongoing across multiple institutions. A Belgian consortium, comprising KU Leuven, the Queen Astrid Military Hospital (QAMH) and Sciensano (Belgium’s Federal Health Agency), has proposed standards based on the practices at the Eliava Institute (9). These require phages to demonstrate an EOP ≥0.1 on a patient’s strain and maintain stable bacterial lysis for 6–48 hours at low multiplicities of infection (MOIs; 0.0001–0.00001 phages per bacterium) at a starting bacterial concentration of 10^6^ CFU/mL. Different criteria have been developed by other institutions: the Polish Academy of Sciences requires >99% killing within 6 hours, while the Center for Phage Technology at Texas A&M considers phages therapeutic candidates based on reproducible plaque formation and stability in physiological conditions (55, 56). However, comparative data evaluating the clinical effect of these varying standards remains limited.

To achieve these standards, phages are often preadapted to patient strains through sequential phage-bacteria coincubation cycles to select the fastest-clearing samples for rapid lysis (57). Adaptations modify genes encoding for receptor-binding proteins and tail fibers, enhancing phage-host interactions. Additional mutations may enhance phage DNA injection, host range, replication, and lysis timing, with specific changes varying by phage-host combination.

## Phage manufacturing

Phage manufacturing involves the production of therapeutic phages for clinical use. It produces high-titer, pure phage preparations that meet safety and potency standards for patient administration. Phage manufacturing consists of three main phases: propagation, purification, and QC (58, 59) (Figure 1A).

### Phage propagation.

Phages require a bacterial host (the “propagation strain”) for multiplication. Key factors for selection of a propagation strain include optimal growth characteristics, absence of lysogenic phages and virulence factors, and the ability to produce consistent high-titer yields. As improved strains can be identified, propagation strains may be updated over time. The propagation process involves inoculating phages into a growing bacterial culture at specific MOIs (10^–5^–10^2^ phages per bacterial cell), with optimal ratios varying by phage type. The culture is then incubated for 4–24 hours in liquid or solid media supplemented with calcium and magnesium to promote phage binding to host bacteria. The resulting lysates undergo centrifugation and filter sterilization, followed by testing to determine the concentration of active phages.

Manufacturing occurs in-house at specialized phage therapy centers or is outsourced (54, 60). Numerous centers, including the Eliava Phage Therapy Center, the Phage Therapy Unit of the Polish Academy of Sciences, the QAMH, Tailored Antibacterials and Innovative Laboratories for phage (Φ) Research (TAILΦR), the Center for Phage Therapy and Biology at Yale, and Phage Australia, operate dedicated microbiology labs for patient-specific phage preparation (9, 33, 61–64). Some facilities, like the Center for Innovative Phage Applications and Therapeutics (IPATH) at UCSD and the Israeli Phage Therapy Center (65, 66), focus on testing and clinical application while outsourcing phage production. Academic research labs also contribute to phage production (67, 68). Most centers produce phages at benchtop scale (~50 mL to 1 L), while some companies use larger bioreactors, such as the Cellexus Cellmaker (4–50 L) (69).

### Phage purification.

Purification is a critical step in preparing phages for safe clinical use (Figure 1A), removing contaminants released during phage replication and bacterial lysis (34). These contaminants, including endotoxins, bacterial nucleic acids, host proteins, and media components, cause severe inflammatory responses (70).

Various purification methods (53, 63, 71) typically begin with nuclease treatment to degrade bacterial DNA and RNA, followed by polyethylene glycol precipitation to eliminate media components and host proteins.

A critical focus of purification is the removal of endotoxins — toxic components of bacterial cell walls that pose the primary safety concern. Multiple approaches have been developed for endotoxin removal, including organic solvent extraction and density gradient ultracentrifugation (72–75). Column chromatography provides automated purification capabilities, but these require specialized equipment, expertise, and phage-specific optimization (76, 77). Following any purification steps, process-introduced chemicals are eliminated via dialysis, filtration, or desalting columns (53). Notably, a recent report demonstrated that simpler methods — combining low-speed centrifugations, microfiltration, and cross-flow ultrafiltration — can effectively reduce endotoxin levels to meet the clinical standard, suggesting complex purification methods involving solvents may be unnecessary for certain phages and applications (53).

### QC.

QC ensures the safety of therapeutic phage preparations. Without phage-specific regulatory guidelines, phage producers often develop internal QC protocols for phage identification, characterization, and purity assessment (34, 70, 78). They generally follow FDA-specified endotoxin limits for all injectable products (5 endotoxin units/kg/h), calculated from the maximum hourly safe dosage using standard formulas (79). QC testing typically adheres to national pharmacopoeia protocols for endotoxin and sterility testing (80). Some jurisdictions, like Belgium, have specific guidelines for more comprehensive QC of phage preparations, including whole-genome sequencing, potency testing, and pH assessment (78). Similar QC protocols are used by phage producers in the United States and Australia. As therapeutic phage applications become more widespread, the field is expected to adopt more standardized and sophisticated purification and QC methods.

## Therapeutic administration

### Routes of administration.

Phage therapy delivery methods are tailored to the patient-specific requirements and site of infection (Figure 1A). While systemic administration involves intravenous (i.v.) delivery, local administration methods vary according to the infection site. Respiratory tract infections use nebulization (81), urinary tract infections may use intravesicular administration (82), prosthetic joint infections need intra-articular delivery (83), and skin infections and wounds use topical applications (60). Local delivery may reach higher phage concentrations at the target site compared with i.v. administration (84–86). Some studies suggest that therapeutic outcomes may be improved through using both systemic and localized delivery methods (12).

### Dosing strategies.

Phage therapy dosing varies in concentration and frequency, ranging from a single dose to multiple daily doses (every 6-, 8-, 12-, or 24-hour intervals) (12, 87). Individual doses typically contain between 10^6^ and 10^10^ plaque-forming units (PFU) (88). The optimal dosing strategy is determined by multiple factors: infection type and severity, phage pharmacokinetics (PK) (including absorption, distribution, and excretion patterns), and accessibility to the infection site (89, 90). For example, respiratory infections need more frequent administration (3–4 times daily) than musculoskeletal infections (once daily) (83, 91). High-dose approaches (>10^9^ PFU/mL) are typically preferred for acute infections requiring rapid bacterial clearance or cases involving poor accessibility or high bacterial loads (92, 93). Lower doses are better suited for chronic infections or scenarios where gradual bacterial reduction is desired (92, 93).

As clinical experience grows and as understanding of phage PK improves, more refined and standardized dosing protocols are expected to emerge (3).

### Therapeutic monitoring.

Treatment safety, efficacy, and patient response are all assessed during monitoring of phage therapy (Figure 1A) (94). The scope and frequency of monitoring are typically determined by the infection site, administration route, and patient’s conditions. Clinical monitoring includes symptoms, physical examinations and vital sign assessments before, during, and after phage administration. Laboratory monitoring uses blood tests for inflammatory markers (e.g., c-reactive protein, erythrocyte sedimentation rate), complete blood count, liver function tests, and basic metabolic panels (64). Additional monitoring may include imaging studies such as X-ray, CT, MRI, or PET scans. Treatment efficacy uses direct monitoring of target bacteria and phages, using bacterial culturing, plaque assays, and/or quantitative PCR (95). This integrated monitoring approach not only ensures patient safety, but also generates valuable data for refining treatment protocols and improving future therapeutic outcomes.

Bacterial resistance to phages can emerge during treatment and may be confirmed through phage susceptibility testing or genome sequencing of resistant isolates (45). This resistance develops through several mechanisms, including modifications to surface receptors, CRISPR/Cas systems, restriction-modification systems, or alterations in membrane transport systems. Importantly, these resistance mechanisms often come with fitness trade-offs that impact bacterial survival and virulence in patients. Such trade-offs can manifest in bacteria as reduced growth rates, increased antibiotic susceptibility, or decreased virulence factor expression (3, 96). Understanding these fitness costs can have important clinical implications, as they may influence treatment outcomes and bacterial persistence, and can inform phage therapeutic strategies. For example, phages have been strategically deployed to select for phage-resistant bacterial populations that show increased antibiotic susceptibility (97).

Throughout and following the treatment course, clinicians carefully monitor patients for both mild and serious adverse events (64). While serious adverse events are rare, documented effects include transient fever and other inflammatory responses after initial doses, localized inflammation at infection sites, and occasional endotoxin-related reactions during Gram-negative bacterial infections (64). Some treatment centers implement immunological monitoring protocols, including measurement of antiphage antibodies and analysis of immune response genes, to better assess patients’ response to phage therapy (95). The immune responses to phage treatment appear to be both phage specific and dependent on the patient’s immune status, with different phages eliciting varying responses — from formation of neutralizing antibodies against phages to secretion of antiinflammatory markers triggered by phages (98, 99).

## Comparative analysis of phage therapy approaches

Phage therapy in clinical settings is primarily deployed through two main approaches: personalized phage therapy and fixed phage therapy (100–102) (Figure 1B). However, recent developments have revealed a more nuanced landscape of phage therapy implementation. In this section, we highlight advantages and limitations of personalized, fixed, and emerging “hybrid” approaches to phage therapy.

### Personalized phage therapy.

Personalized phage therapy involves selecting phages to target the specific bacterial strain(s) responsible for a patient’s infection (11, 12, 15, 65–72) (Table 1). This approach is typically implemented at a “phage therapy center,” which often constitutes academic-medical institutions providing phage treatments to patients primarily on a compassionate use basis. Some examples include the Eliava Phage Therapy Center, the Phage Therapy Unit of the Polish Academy of Sciences, QAMH, the Center for Phage Biology and Therapy at Yale, TAILOR, IPATH, the Israeli Phage Therapy Center, Phage Australia, and the Mayo Clinic Phage and Lysins Program.

Personalized phage therapy requires extensive screening of phage libraries and/or environmental samples, coupled with phage preadaptation to infection conditions (4, 63, 103–106). This approach often involves iterative cycles of phage testing and preparation to address phage-resistant bacterial isolates, and most centers employ therapeutic monitoring during treatment. While clinical outcomes have been promising, with reported improvement rates of 77.2% in treated cases (8, 9), the approach faces several challenges, including lack of standardization, time-consuming patient-specific preparation protocols (limiting utility in acute cases), and regulatory ambiguity. In the United States, treatments are conducted through the FDA’s emergency investigational new drug (eIND) program, which requires comprehensive documentation of phage preparation, safety testing, and treatment rationale. Some institutions have established FDA master files to streamline this process. Despite encouraging case reports and studies, controlled clinical efficacy trials using the personalized approach have yet to be published (8, 9, 16).

### Fixed phage therapy.

Fixed phage therapy uses preformulated phage preparations, often as phage cocktails, designed to target a broad range of bacterial species (107–110) (Table 1). This approach aligns with traditional biologic drug development pathways, offering advantages of standardized, large-scale production that reduces per-patient costs and simplifies logistics (109, 111). Development of these cocktails involves strategic phage selection to maximize therapeutic coverage, including targeting diverse bacterial receptors and using data-driven approaches to identify phages with complementary host ranges (40, 111–113).

Fixed phage cocktail trials have shown limited success to date. A recent systematic review revealed that only two of seven efficacy trials demonstrated therapeutic success (114). This approach faces several inherent challenges. First, the need to predict target pathogens in advance affects both product development and clinical implementation. Most fixed cocktails target only a single bacterial species — primarily *Staphylococcus aureus* or *Pseudomonas aeruginosa* — despite at least 30 different bacterial species being involved in difficult-to-treat infections. This narrow targeting creates recruitment challenges and affects trial efficacy when actual infections do not match cocktail specificity (9, 60, 115, 116). Additional technical hurdles include maintaining therapeutic phage concentrations during long-term storage and distribution of premade cocktails. Current trials are attempting to address these limitations through improved design strategies, such as incorporating preliminary bacterial susceptibility screening phases. However, more rigorously designed trials are needed to properly evaluate the potential of fixed phage therapy (16, 60, 115–119).

### Emerging hybrid models.

Hybrid models have emerged that combine key strengths of both personalized and fixed phage therapy approaches. For example, centers producing personalized phage preparations have begun to administer the same phage preparations to multiple patients, while still often performing the patient-specific phage susceptibility testing, analysis of phage-resistant mutants, and/or therapeutic monitoring that is characteristic of the “personalized” approach (9, 62, 66, 120). This strategy can bring the economies of scale and streamlined logistics of preprepared cocktails without sacrificing the benefits of the personalized approach.

However, integrating phage therapy into the current regulatory framework for licensed medicinal products presents significant challenges. Traditional pharmaceutical regulations, designed for static drug products, are poorly suited to accommodate phage therapy’s dynamic nature, particularly the need for rapid updates to counter bacterial evolution. Several key regulatory hurdles exist: the requirement for extensive premarket safety and efficacy data from large clinical trials is especially challenging for such a targeted therapeutic, while current manufacturing standards and QC requirements are difficult to satisfy given the biological complexity and natural variation inherent in phage products. Moving forward, new regulatory frameworks may be necessary, potentially drawing inspiration from existing models used for other complex biological products, such as fecal microbiota transplants, blood safety protocols, and the annual updating process for seasonal flu vaccines.

## Gaps in phage therapy development

**Figure 2:**
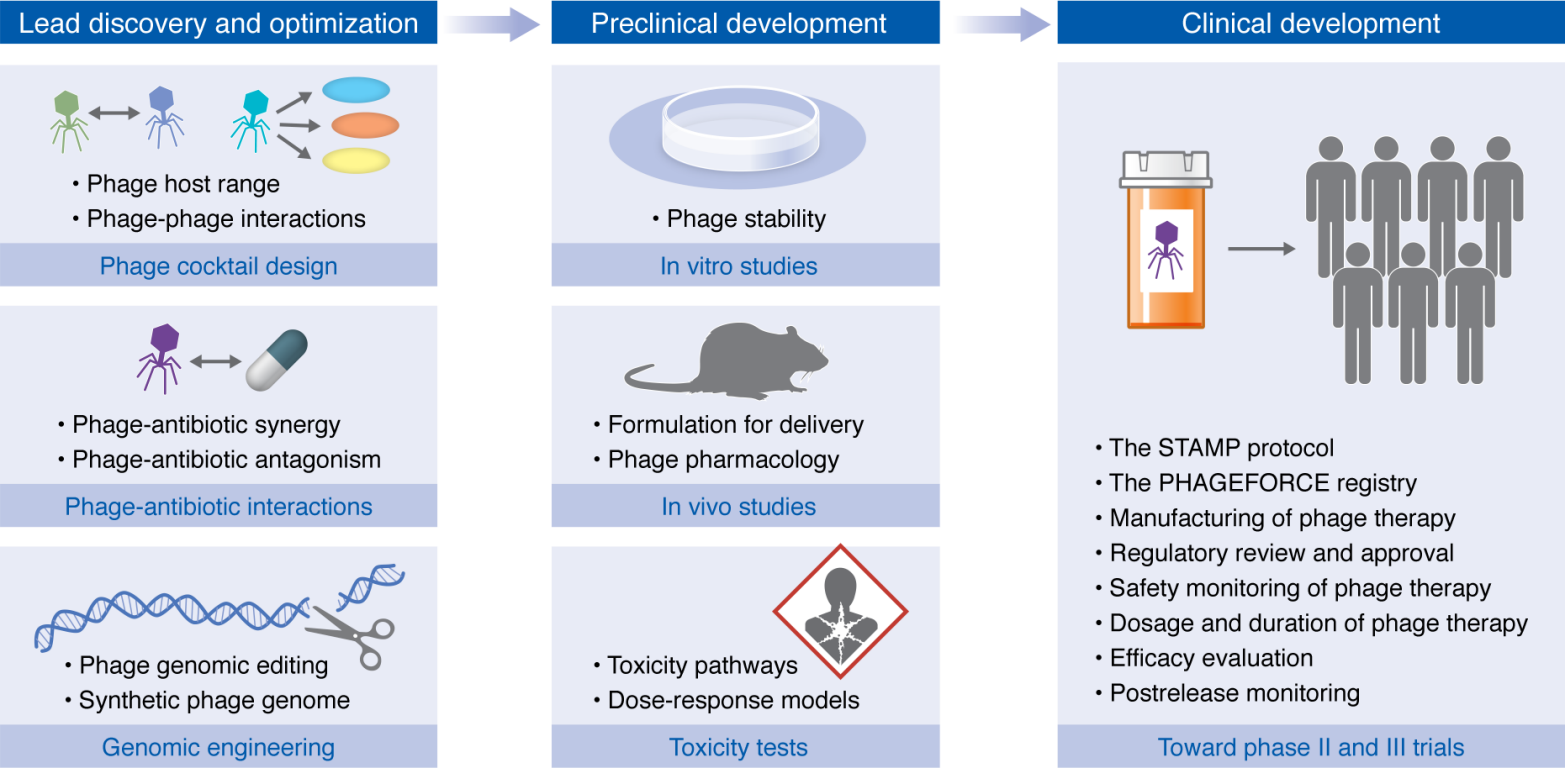
Gaps in phage therapy through the perspective of a drug development pipeline. The drug development pathway consists of three major phases: lead discovery and optimization, preclinical development, and clinical development. In lead discovery and optimization, key areas requiring further research include phage cocktail design (understanding phage host range and phage-phage interactions), phage-antibiotic interactions (investigating both synergistic and antagonistic effects), and genomic engineering (developing phage genomic editing techniques and synthetic phage genomes). Preclinical development encompasses in vitro studies (focusing on phage stability), in vivo studies (addressing formulation for delivery and phage pharmacology), and toxicity tests (evaluating toxicity pathways and dose-response models). The clinical development phase involves multiple critical components: establishment of manufacturing processes, regulatory review and approval procedures, safety monitoring protocols, optimization of dosage and duration regimens, efficacy evaluation, and postrelease monitoring. Addressing these knowledge gaps will be necessary for successful implementation of clinical phage therapy and to broaden applications for phage-based strategies.

Despite advances in phage therapy, substantial knowledge gaps persist. These challenges may best be understood through the lens of a drug development pipeline, which includes lead discovery and optimization, preclinical development, and clinical development (Figure 2).

## Lead discovery and optimization

### Phage cocktail design.

Designing optimally effective phage cocktails remains a considerable challenge in phage therapy development. Phage-phage interactions can be synergistic or antagonistic, species dependent, and difficult to predict. The optimal number and ratio of phages in a cocktail is unclear, and standardized protocols for interrogating phage-phage combinations are lacking. Consequently, phage cocktails are often selected empirically (116, 121).

Several models for phage cocktail design exist (112), including strain-based and genomic algorithms (108, 122). Strain-based algorithms use analysis of host range data across large bacterial strain collections and prediction of minimum phage combinations providing maximum strain coverage. Genomic algorithms incorporate additional layers of analysis, such as evaluation of bacterial receptor genes and prediction of phage-host interactions based on receptor recognition patterns, and then assessment of potential resistance mechanisms through genome mining. These computational approaches can be used individually or in combination to optimize cocktail composition. Alternative approaches include experimentally matching phages to each individual bacterial strain in a collection (123–125). However, scaling up these approaches to encompass the vast diversity of bacteria in clinical settings is challenging.

Bacterial receptors play a crucial role in determining phage host range (40), and theoretically, creating cocktails that target all possible bacterial receptor specificities could provide broad coverage. Cocktails containing phages using different receptors have explored this strategy (113), though they have typically been limited to a few strains and have not consistently achieved bacterial eradication. Challenges regarding cocktail design include insufficient coverage of receptor types, emergence of cross-resistance between phages, and inadequate phage concentrations to prevent resistant subpopulations from emerging (108). Recent attempts combining phages targeting multiple nonredundant receptors have been successful in biofilms and in an animal wound infection model against large numbers of diverse clinical isolates of *P*. *aeruginosa* and *S*. *aureus* (111). While this approach offers a promising direction for future phage cocktail design, some bacterial species may still develop resistance. For some species, exploiting trade-offs associated with phage resistance, such as reduced virulence or antibiotic resensitization, may thus be necessary alongside cocktail design strategies (3).

### Phage-antibiotic interactions.

Notable gaps remain in optimizing phage-antibiotic interactions for clinical use. Some phages act synergistically with antibiotics (8, 117, 126, 127). Some antibiotics enhance phage activity at subinhibitory concentrations (87, 128, 129), while some can completely suppress phage resistance development at high concentrations (127). Phages can also resensitize antibiotic-resistant bacteria by targeting resistance mechanisms such as efflux pumps or outer membrane components as receptors (9, 97, 130–132). However, some antibiotics, particularly protein synthesis inhibitors, can antagonize phage activity by interfering with phage replication (133). The specific pairing of phage and antibiotic is challenging to predict but crucial for optimizing treatment efficacy (109, 127).

Both personalized and fixed phage therapy often incorporate combination therapy with antibiotics to enhance efficacy and mitigate resistance development (126–128, 134). In vitro assessment of phage-antibiotic synergy is a common practice to guide combination therapy (135), and successful outcomes using this approach have been reported in several studies (136). For instance, in a study of 100 cases employing personalized phage therapy, phages were deployed alongside antibiotics in approximately 70% of cases, resulting in great outcome (9). Further research is needed to understand the long-term phage-antibiotic-bacterial dynamics and develop predictive models for optimizing phage-antibiotic therapy in clinical settings.

### Phage genome engineering.

Wild-type phages demonstrate therapeutic potential (137) but have challenges, including narrow host ranges, lysogenic conversion, immunological clearance, and variable stability (87). To overcome these, researchers use genetic engineering approaches. Recent progress focuses on two approaches: editing phage genomes and synthesizing new ones (4, 138). For genome editing, CRISPR/Cas systems and methods like BRED (Bacteriophage Recombineering of Electroporated DNA) have been developed (139–143). Production of synthetic phage is also advancing rapidly toward the goal of chemical synthesis of entire phage genomes in bacteria or cell-free systems (35, 144, 145). This synthetic approach could markedly improve scalability and safety by eliminating bacterial components from the manufacturing process.

The regulatory landscape for engineered phages varies by jurisdiction. In the United States, engineered phages fall under FDA oversight as biological products, while the European Medicines Agency considers them Advanced Therapy Medicinal Products. Several engineered phages have been successfully proceeded through eIND provisions, including modified lysogenic phages with deleted lysogeny genes and phages engineered for enhanced stability or biofilm degradation (146). However, owing to safety considerations, regulatory frameworks generally favor strictly lytic phages for therapeutic applications over lysogenic or engineered phages (147).

The future of phage engineering will likely focus on both optimizing therapeutic applications and expanding into new frontiers, including targeted delivery of gene editing payloads and microbiome modulation (4). Advances in DNA synthesis will enhance flexibility in designing synthetic phages, improving properties like efficacy, stability, delivery, and safety profiles (144). Additionally, generative AI models trained on phage genomic sequences (148) open new possibilities for designing and synthesizing phages with desired properties from scratch. However, successful implementation of these approaches will still require in-depth understanding of phage biology (149), and thus continued research will remain crucial for advancing phage engineering.

## Preclinical development

### Phage stability.

Substantial gaps remain in controlling phage stability, which encompasses titer in solution and physical integrity over time. Basic principles include stability at physiological pH (150–152) and the importance of cations for stability and activity (153–156). However, many factors contributing to stability loss are poorly understood and phage specific. Phages are commonly formulated in buffered, cation-supplemented saline solutions (157), but various factors can reduce phage titer over time. These include adsorption to surfaces (e.g., storage containers, catheters) (158) and interactions with bacterial components such as lipids, membrane debris, or vesicles (159–161). Some phages are more stable when purified, while others maintain better stability in lysates, highlighting the need for phage-specific optimization.

Physical factors impact phage stability, including temperature extremes that cause denaturation, aggregation, or structural loss (162–165). Oxidative stress creates aggregates and fragments (166–169), while UV light exposure degrades phage particles (163, 170). Common mitigation strategies include controlled temperatures, cryoprotectants, and UV-protective additives (171). The phage-specific nature of these environmental stressors highlight the challenges in developing universally effective storage protocols.

Phage stability is measured through plaque assay titers and qPCR. However, these methods do not capture physical changes like aggregation or degradation. Recent advancements, such as using dynamic light scattering, offer new ways to rapidly assess changes in phage bioactivity (163), but more work is needed to develop comprehensive, standardized stability assessment methods across diverse therapeutic applications.

### Phage formulation for clinical applications.

While clinical applications of phage formulations show safety (105, 172–175), crucial gaps persist in optimizing formulations for diverse administration routes and clinical scenarios.

For systemic administration, phages are often reconstituted in saline or pH-balanced buffers (83, 176–178), though optimal formulation varies by infections. Recent advances in formulation technologies, particularly spray-drying, show promise for enhancing stability and shelf-life (148), offering improved solutions for storage, transport, and administration.

Oral phage therapy may necessitate protection from stomach acid, using encapsulation or coadministration with pH-raising additives (93, 179, 180). Animal studies demonstrate improved bioavailability when phages are coadministered with agents that overcome the stomach acid barrier (181). Notably, a diverse range of formulation methodologies has emerged, including microencapsulation, nanocarriers, and advanced polymer-based delivery systems (182). However, formulations ensuring consistent oral bioavailability are yet to be determined.

Wound phage therapy has primarily relied on two approaches: topical solutions or phage-impregnated dressings, albeit with variable efficacy (183–186). For respiratory applications, delivery options include nebulized suspensions, dry powders, and soft mist inhalers, with dry powder formulations offering improved half-life (187) and soft mist inhalers providing superior lung delivery (188).

Preclinical studies are exploring various excipient strategies, including ionic hydrogels, microparticles, and liposomes for rapid burst-release, while fibrin glue and dynamic covalent cross-linked hydrogels enable extended-release dynamics (189–197). Despite these advances, further research is needed to optimize phage formulations to maximize therapeutic benefit while maintaining safety across different administration routes and infection types.

### Phage pharmacology.

Understanding the PK and pharmacodynamics (PD) of phages is crucial for optimizing therapeutic efficacy in clinical settings (93, 177, 198). However, achieving a comprehensive understanding of PK/PD for phage therapy is challenging owing to the complex three-way interactions between phages, bacteria, and the human host. Since nearly every phage-bacteria-patient combination may exhibit a unique PK/PD profile, developing standardized models applicable across diverse clinical scenarios remains challenging.

PK in phage therapy involves studying the absorption, distribution, metabolism, and excretion of phages in the body (199, 200). Administration routes present distinct challenges: oral administration must overcome gastric conditions (201), while i.v. delivery faces potential clearance by the reticuloendothelial system (202, 203). The role of host immune status in phage PK is emerging as an important consideration, providing insights into phage-immune interactions emerging from recent studies (99, 204). Mouse models have shown that immune status can significantly impact phage therapy effectiveness (205, 206), suggesting that immunocompromised hosts may experience prolonged phage circulation times, which could potentially enhance therapeutic effects. Phage-immune interactions also affect therapeutic outcomes differently in acute versus chronic infections (206). Understanding these complex pharmacokinetic processes and immune-phage interactions is crucial for optimizing phage therapy efficacy and safety.

Phage PD, which describes the interaction between phages and their bacterial targets in vivo (92, 207), remains poorly understood. A key challenge is assessing the MOI in vivo, which is known to be important in vitro but nearly impossible to assess in patients due to uncertainties in bacterial load at the infection site. This gap necessitates systematic studies to understand the relationship between MOI, killing efficiency, and resistance development (195).

Modeling PK/PD for phage therapy is further complicated by the ability of phages to replicate at infection sites, unlike traditional antibiotics. Comprehensive models are needed that account for phage replication and bacterial population dynamics. Additionally, standardizing phage measurement techniques, such as plaque assays and qPCR, is crucial for accurately determining PK/PD parameters across different studies and clinical scenarios.

## Clinical development

### Clinical trial design.

It is widely acknowledged that controlled clinical trials are needed to demonstrate phage therapy efficacy. Past phage therapy clinical trial failures are largely attributed to trial design issues (as described in *Fixed phage therapy*). As a result, the clinical efficacy of phage therapy has not yet been fully evaluated for any indication.

Encouragingly, multiple organizations are now funding randomized controlled trials. The US Department of Defense, NIH, and biotechnology companies are investigating phage therapy for various conditions, including diabetic foot ulcers, respiratory infections, prosthetic joint infections, and urinary tract infections (208, 209). Preliminary results from these trials show promise.

New innovative nonrandomized trial designs have also emerged to collect data from personalized phage therapy treatments worldwide, while informing future controlled trial designs. For example, Phage Australia’s STAMP (Standardized Treatment and Monitoring Protocol) study uses an open-label, single-arm design to assess safety, tolerability, and feasibility of phage therapy across multiple centers, pathogens, and clinical indications (63). This allows for flexible, patient-specific phage matching while maintaining consistent dosing and monitoring across patients. Similarly, the PHAGEFORCE registry at UZ Leuven in Belgium offers a prospective, observational approach comparing phage therapy outcomes against standard of care (210). In this design, patients receive phage therapy with standard care when active phages are available; otherwise, they form the control groups receiving standard of care alone. This diverse range of ongoing trials demonstrates the field’s momentum toward establishing phage therapy in modern clinical practice, while innovating on past approaches to finally evaluate if, when, and how phage therapy can be efficacious in the clinic.

Phage therapy is not alone in requiring innovations on traditional clinical trial design to demonstrate efficacy. CAR T cell therapy has successfully demonstrated efficacy for personalized cancer treatments despite patient-specific requirements (211). Palliative care research has employed “*n* of 1 trials” to address challenges in patient recruitment and high interpatient variability (212). Although these approaches could inform phage therapy trial designs, the distinctive economic challenges in antimicrobial development may necessitate further innovations to balance scientific rigor with cost-effectiveness in clinical trials.

## Conclusion

The need for therapeutics against MDR infections is growing, and the field of phage therapy is rapidly advancing to meet this challenge. In recent years there has been substantial refinement in approaches for phage selection, production, and delivery. Improvements in phage technology are enabling personalized phage therapy, while advancements in AI and bioengineering seem poised to create substantial therapeutic and commercial opportunities.

Nonetheless, numerous challenges remain. While the general steps required for successful clinical phage therapy implementation are becoming clearer, widespread availability still depends on addressing key challenges across all approaches. These include optimizing phage cocktail design, standardizing phage susceptibility testing, developing PK/PD methods, and improving stability and formulation. Determining optimal parameters for specific clinical indications while reducing preparation time will be critical in improving outcomes and broadening the applicability. Many acute infections like sepsis are extremely time sensitive, which may limit the applicability of personalized phage therapy. Chronic infections often involve biofilms, which can limit phage efficacy and are not well accounted for in standard susceptibility testing. Nonetheless, despite these challenges, reported clinical benefits still have exceeded 70% in treated cases in several recent series.

While we are encouraged by the recent progress in the field, it is clear that a drug development pipeline for phage therapy is needed and that this is likely to emerge only with government support. Fortunately, several national governments, including those of Belgium, Australia, the United States, and Great Britain, have recognized the promise of phage therapy and have contributed to bringing it to its current state. However, given the broken economics of antimicrobial development, increased government involvement through direct funding and regulatory changes is needed. Legislation like the proposed PASTEUR Act, which would authorize the US government to enter into subscription contracts for critical-need antimicrobials, as well as provide $6 billion in funding, could support this pipeline. Such initiatives could provide the necessary incentives for drug developers to invest in phage therapy development, ultimately renewing our arsenal against infectious diseases for future generations.

## Figures and Tables

**Table 1:**
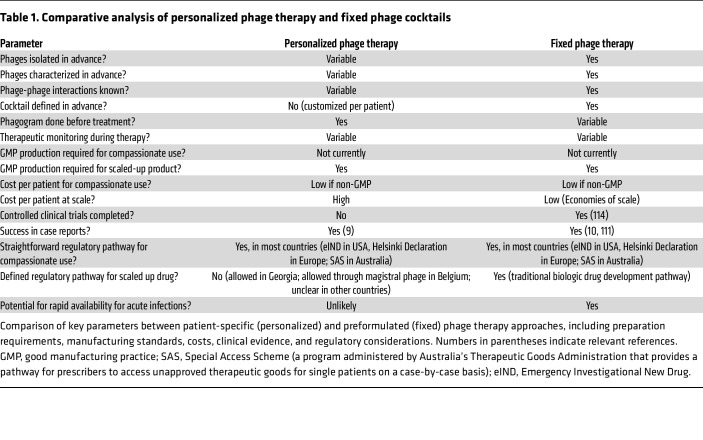
Comparative analysis of personalized phage therapy and fixed phage cocktails

## Version 1 03/03/2025

Electronic publication
